# Isolated Spontaneous Renal Artery Dissection Presented with Flank Pain

**DOI:** 10.1155/2015/896706

**Published:** 2015-05-18

**Authors:** Shruti P. Gandhi, Kajal Patel, Bipin C. Pal

**Affiliations:** ^1^Department of Radiology and Imaging, G. R. Doshi and K. M. Mehta Institute of Kidney Diseases and Research Centre (IKDRC) and Dr. H.L. Trivedi Institute of Transplantation Sciences (ITS), Gujarat University, Ahmedabad 380016, India; ^2^Department of Urology, G. R. Doshi and K. M. Mehta Institute of Kidney Diseases and Research Centre (IKDRC) and Dr. H.L. Trivedi Institute of Transplantation Sciences (ITS), Gujarat University, Ahmedabad 380016, India

## Abstract

Spontaneous renal artery dissection is a rare but important cause of flank pain. We report a case of isolated spontaneous renal artery dissection in 56-year-old man complicated by renal infarction presented with flank pain. Doppler study pointed towards vascular pathology. Computed tomography (CT) angiography was used to make final diagnosis which demonstrated intimal flap in main renal artery with renal infarction.

## 1. Introduction

Flank pain is a common complaint in the emergency and general urology which might be caused by a variety of urinary and extraurinary abnormalities. Among these urolithiasis is the most frequent cause. It is suggested that at least half of patients with acute flank pain have no evidence of stone disease on computed tomography (CT), and an alternative diagnosis is often found [1]. Spontaneous renal artery dissection (SRAD) is a rare and serious event that can result in renal parenchymal injury and severe hypertension. The true incidence, etiology, and natural history of this phenomenon are not precisely defined. Diagnosis is often delayed due to difficulties in distinguishing the clinical presentation from more familiar conditions. Ultrasound and Colour Doppler examination is not sensitive enough to detect the dissection or renal infarction. We report a case of SRAD in a healthy 56-year-old male who presented with flank pain. Colour Doppler study failed to demonstrate dissection flap but suggested vascular etiology. Computed tomography angiography (CTA) was used to make final diagnosis which showed renal infarction with intimal flap in main renal artery. The use of high resolution CTA has obviated the need for femoral arteriography.

## 2. Case Presentation 

A 56-year-old male presented to our institute with history of right sided flank pain since morning. The pain was constant in nature and associated with nausea and vomiting. Patient had no history of dysuria, haematuria, or fever. Blood pressure at time of presentation was 180/110 mmHg and heart rate was 90 bpm. His cardiovascular history was unremarkable except that he is hypertensive and he was on regular treatment with single antihypertensive drug, calcium channel blocker tablet nifedipine 20 mg since 2 years and his blood pressure was remaining within normal limits. Electrocardiography was normal. His abdomen was soft, nontender, and nondistended. Bowel sounds were normal. He had past history of radical left nephrectomy for renal cell carcinoma three years back. Patient had no history of diabetes, dyslipidaemia, or smoking. There was no history of surgery, trauma, or any instrumentation in right kidney. Blood and urine samples were collected for analysis and patient was referred to department of radiology and imaging with presumed diagnosis of urolithiasis.

**Figure 1:**
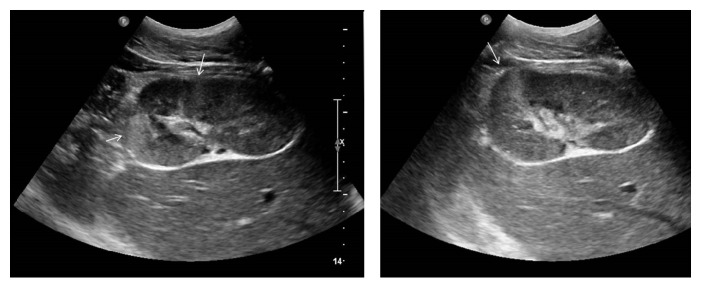
Findings: grey scale ultrasound image of right kidney showing ill-defined echogenic areas at lower pole of right kidney.

**Figure 2:**
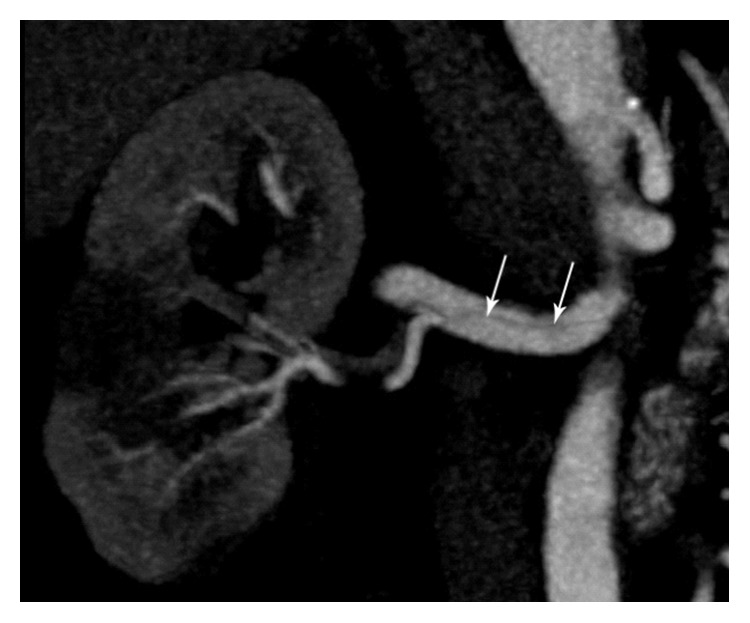
Findings: maximum intensity projection, in coronal plane of CT renal angiography in arterial phase demonstrating linear filling defect in main renal artery, suggests intimal flap (long arrow).

**Figure 3:**
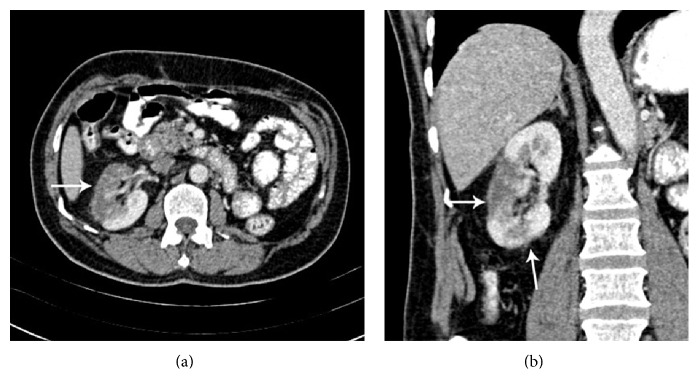
Findings: axial (a) and coronal (b) image of CT angiography in venous phase showing nonenhancing wedge shaped areas at mid and lower pole (arrows) of right kidney suggestive of infarcts.

Plain X-ray of kidney ureter bladder (KUB) was normal. Abdominal ultrasound and noncontrast CT KUB showed no evidence of calculus or hydronephrosis. There were ill-defined echogenic areas found at mid and lower pole of right kidney in grey scale ultrasound (Figure 1) which were reported as possible changes of pyelonephritis, subjected to pending laboratory investigation. However, blood analysis showed normal white cell count, haemoglobin, and electrolytes. His serum creatinine was 1.6 mg/dL (normal: 0.6–1.2 mg/dL). Urine analysis was negative with no evidence of pus cells or red blood cells in urine. Patient was referred again to radiology department to review ultrasound findings. While scanning the right kidney, on putting colour Doppler, no evidence of intrarenal perfusion was found in mid and lower pole suggestive of infarction. Upper pole of kidney showed normal perfusion with 0.68 resistive index (normal: 0.60 to 0.70) and 0.07 sec acceleration time (normal: <0.07 sec) on spectral analysis. There was no evidence of renal vein thrombosis. However, full length of main renal artery could not be seen due to obesity and bowel gas. Origin of right renal artery was seen in banana peel view (coronal view through liver window) and it showed 110 cm/sec peak systolic velocity (normal: <120 cm/sec) at origin. As Doppler finding pointed towards vascular pathology CT angiography was carried out on Somatom Sensation 64 CT scan. CT angiography showed an intimal flap in right main renal artery with double lumen (Figure 2). There was evidence of nonenhancing wedge shaped areas at mid and lower pole of kidney suggesting infarctions (Figure 3). All other abdominal arteries and organs were unremarkable.

## 3. Discussion

Renal artery dissection can be acute or chronic. Acute renal artery dissection is divided into three types: iatrogenic (guide wire, catheter, and angioplasty balloon), spontaneous, and agonal (sepsis, malignancy, stroke, chronic renal failure, and cirrhosis). Chronic renal artery dissection is classified as functional and silent [2]. Spontaneous renal artery dissection (SRAD) is a rare but important cause of flank pain, and clinician should be mindful of it as a differential diagnosis. It may be both the aetiology and consequence of uncontrolled hypertension. Sustained elevated blood pressure may contribute to the development of arterial dissection by potentiating arteriosclerosis and medial degeneration [3], although hypertension itself may be secondary to renal ischemia or severe pain following the SRAD. Depending on the severity, extent, and main artery versus branch involvement, SRAD may cause renal ischemia of varying degree, renin-mediated renovascular hypertension, and renal infarction [4–7]. Other conditions associated with the development of SRAD include fibromuscular dysplasia, malignant hypertension, severe atherosclerosis, Marfan syndrome, Ehlers-Danlos syndrome, subadventitial angioma, polyarteritis nodosa, and cystic medial necrosis [8–11]. Cocaine abuse and extracorporeal shock wave lithotripsy were reported in rare cases [12, 13]. Unusual physical stress can lead to traction of renal arteries and abnormalities in the integrity of connective tissue may present predisposing factors that can result in rupture of vasa vasorum and subsequent cascade of events such as intramural hematoma and SRAD [11]. Since our patient had no history of renal colic, trauma, or instrumentation in the past and his right kidney was reported as normal in previous contrast enhanced CT done 3 years back for evaluation and staging of left renal carcinoma, the dissection was classified as acute and spontaneous.

Most patients are male (a 4 : 1 ratio), in their 4th-5th decade of life [6]. The male predominance could be even stronger with a ratio of 10 : 1 as described by Edwards et al. [14]. It has no predilection for either side; it may be bilateral in 10–15% of cases [15–17]. Spontaneous renal artery dissection may be accompanied by dissection of other peripheral arteries [18]. In cases of spontaneous renal artery dissection with renal infarction, the diagnosis must be made as early as possible to increase the chances of renal revascularization and recovery.

Clinical manifestations vary from minimal symptoms to life-threatening hypertension and/or renal failure. The most common symptom of SRAD is intense lumber pain or flank pain [15]. Pain may radiate to the epigastrium or in inguinal region. The most common sign on clinical examination is hypertension [2]. Other clinical presentations include haematuria, proteinuria, abdominal bruits, and acute renal failure. Because of this vague clinical presentation, it may simulate urolithiasis or pyelonephritis and SRAD can only be confirmed by imaging.

Renal ultrasound and Doppler have poor diagnostic sensitivity. The utility of intravascular ultrasound is well described in the literature for determining the false and true lumens in aortic dissections [19], although there is very limited information on intravascular ultrasound in the evaluation of visceral/renal artery dissection. A use of intravascular ultrasound for diagnosis of renal artery dissected is reported by Watanabe et al. [20]. Renal isotopes study may detect renal infarction. Angiography is a gold standard for diagnosis. CT angiography is noninvasive, faster, and easily available so it is a better choice compared to conventional or MR angiography. CT angiography also precisely indicates extent and nature of vascular disease. Dissections and thromboemboli appear differently on angiography; the dissection appears as linear filling defect in lumen of artery or as uniform narrowing due to nonfilling of the false lumen, whereas the emboli display a meniscus crossing the width of the artery [21].

In this case, Doppler ultrasound fails to demonstrate intimal flap. However, the diagnosis of renal infarction was facilitated by ultrasound and colour Doppler, which ultimately demands detailed evaluation of renal artery vasculature by CT angiography. Thus it provides road map to the diagnosis. Renaud et al. reported 6 cases of SRAD with renal infarction; ultrasound failed to detect renal infarction in all cases [22].

As a consequence of the rarity of spontaneous renal artery dissection, there is limited experience regarding its management or natural history. Treatment options include observation, medical management with anticoagulation, control of hypertension, and close follow-up if renal function and blood pressure are stable [15]. Endovascular intervention with stenting or coiling, or surgical revascularisation, may be required if repeat angiography shows an unstable lesion or if hypertension remains uncontrollable with maximal medical therapy. In addition, acute deterioration of renal function also requires intervention [15]. Overall, with the ongoing advances, angioplasty is favourable to surgical revascularization as it is less invasive, less expensive, and associated with a lower morbidity. However, surgical revascularization is indicated in kidneys with substantial residual renal function. Primary nephrectomy should be considered if the kidney is already severely damaged due to infarction and has poor function on isotope renography or if revascularization would be difficult because of renal branch artery involvement [23, 24]. Finally, skilled vascular surgeons can also operatively repair the dissection. This can be done either in situ or ex vivo and requires expertise. In situ repair is more feasible if the dissection does not involve the main renal artery and ex vivo repair is more practical in dissections involving the main renal artery. Although this procedure can achieve satisfactory results, a number of postoperative complications including fibrotic stenosis, thrombosis, partial atrophy, and renal failure can ensue [23]. Extracorporeal reconstruction and autotransplantation were also reported in patients with spontaneous renal artery dissection located in or extending into the distal branches [5]. For the management of this case, a cardiology and urology opinion was sought. Since this patient had solitary unit with marginally raised creatinine, patient was kept on conservative medical management.

There are also some documented cases of SRAD leading to a spontaneous resolution. This can be due to reentry of the false lumen back into the true lumen or due to a complete obliteration of the dissected segment by thrombosis and organization [17]. The rare complication of dissection is an arterial rupture. Two cases have been reported where such a dissection did not result in mortality [6]. The main cause of mortality in patients with SRAD is renal failure. The chances of SRAD being fatal have been shown to increase with bilateral lesions [2].

## 4. Conclusion 

Spontaneous renal artery dissection is a rare but important cause of flank pain and often presents as a diagnostic and therapeutic challenge. Acute flank pain without urolithiasis on noncontrast CT should warrant the physician to carry out quick and detailed work-up and early diagnosis to prevent serious consequences. The diagnosis of SRAD should be advocated when renal crisis is not associated with urolithiasis or alternatively when hypertension is present. Doppler sonography has poor diagnostic sensitivity for definite diagnosis but it may provide road map to diagnosis. CT angiography, rather than conventional angiography, is the gold standard for diagnosis.

## Abbreviations

SRAD: Spontaneous renal artery dissection
CT: Computed tomography
KUB: Kidney ureter bladder.
